# The Role of IL-17 in the Pathogenesis of Oral Squamous Cell Carcinoma

**DOI:** 10.3390/ijms24129874

**Published:** 2023-06-08

**Authors:** Nevena Ladjevac, Marija Milovanovic, Andra Jevtovic, Dragana Arsenijevic, Bojana Stojanovic, Milica Dimitrijevic Stojanovic, Bojan Stojanovic, Nebojsa Arsenijevic, Aleksandar Arsenijevic, Jelena Milovanovic

**Affiliations:** 1 Department of Otorhinolaryngology, General Hospital Uzice, 31000 Uzice, Serbia; dr.nevena.ladjevac@gmail.com; 2 Center for Molecular Medicine and Stem Cell Research, Faculty of Medical Sciences, University of Kragujevac, 34000 Kragujevac, Serbia; marijaposta@gmail.com (M.M.); andrajevtovic@gmail.com (A.J.); menki@hotmail.com (D.A.); bojana.stojanovic04@gmail.com (B.S.); milicadimitrijevic@yahoo.com (M.D.S.); bojan.stojanovic01@gmail.com (B.S.); nebojsa_arsenijevic@yahoo.com (N.A.); 3 Department of Microbiology and Immunology, Faculty of Medical Sciences, University of Kragujevac, 34000 Kragujevac, Serbia; 4 Department of Otorhinolaryngology and Maxillofacial Surgery, Faculty of Medical Sciences, University of Kragujevac, 34000 Kragujevac, Serbia; 5 Department of Pharmacy, Faculty of Medical Sciences, University of Kragujevac, 34000 Kragujevac, Serbia; 6 Department of Pathophysiology, Faculty of Medical Sciences, University of Kragujevac, 34000 Kragujevac, Serbia; 7 Department of Pathology, Faculty of Medical Sciences, University of Kragujevac, 34000 Kragujevac, Serbia; 8 Department of Surgery, Faculty of Medical Sciences, University of Kragujevac, 34000 Kragujevac, Serbia; 9 Department of Histology end Embryology, Faculty of Medical Sciences, University of Kragujevac, 34000 Kragujevac, Serbia

**Keywords:** interleukin-17 (IL-17), oral squamous cell carcinoma (OSCC), inflammation, tumor progression, therapeutic target

## Abstract

Elucidating the inflammatory mechanisms underlying formation and progression of oral squamous cell carcinoma (OSCC) is crucial for discovering new targeted therapeutics. The proinflammatory cytokine IL-17 has proven roles in tumor formation, growth, and metastasis. The presence of IL-17 is demonstrated in both in vitro and in vivo models, and in OSCC patients, is mostly accompanied by enhanced proliferation and invasiveness of cancer cells. Here we review the known facts regarding the role of IL-17 in OSCC pathogenesis, namely the IL-17 mediated production of proinflammatory mediators that mobilize and activate myeloid cells with suppressive and proangiogenic activities and proliferative signals that directly induce proliferation of cancer cells and stem cells. The possibility of a potential IL-17 blockade in OSCC therapy is also discussed.

## 1. Introduction

Among all head and neck carcinomas, including tumors arising from epithelial surfaces from the oral cavity, pharynx, larynx, and paranasal sinuses, and major and minor salivary glands, the most common are oral cell carcinomas representing almost 50% of such cases. The most frequent malignancy among oral cell carcinomas is oral squamous cell carcinoma (OSCC) accounting approximately 90% of these cases [1,2]. The OSCCs are tumors that arise in the oral cavity with localization on the lips, gums, lining of the cheeks and lips, front two-thirds of the tongue, floor of the mouth under the tongue, roof of the mouth, and oropharynx [3]. The primary approaches for the treatment of OSCC are traditional surgery, radiotherapy, and a combination of surgery and radiotherapy that, although it has been improved in recent years, has failed to increase the survival. The global 5-year survival rate is about 50% of all OSCC cases [4]. Prediction of survival in OSCC depends on classical parameters including tumor grade, depth of invasion, the time of diagnosis, and the inflammatory score, thus early-stage OSCC patients have a 5-year survival rate of about 75%, while patients with advanced stages of OSCC at diagnosis have only a 35% survival rate [5,6]. Immunotherapy has been recently introduced as an effective treatment option for OSCC and was firstly approved for recurrent/metastatic cases [7], and for preoperative neoadjuvant immunotherapy for untreated OSCC [8].

Discovering the mechanisms that promote malignant transformation, tumor progression, invasion, and metastasis are fundamental in the process of searching for new targeted therapeutics. There is plethora of evidence regarding an association between OSCC and chronic inflammation [9,10]. Chronic inflammation is a common feature of established OSCC [11]. Dysregulation of different genes whose products are involved in inflammation, wound healing, and angiogenesis was discovered in OSCC cell lines and tissue samples obtained from OSCC patients by microarray analysis [9]. Inflammatory and immune cells that are the main components of the tumor microenvironment affect the processes of OSCC proliferation, survival, invasiveness, and metastasis [12]. Furthermore, the progressive increase in density of inflammatory infiltration has been detected in parallel with increasing grades of oral malignant transformation process from non-dysplastic hyperkeratosis, epithelial dysplasia to OSCC [12,13]. Chronic oral inflammatory conditions, oral lichen planus, submucous fibrosis, and oral discoid lupus, are all predisposing for the development of OSCC [14]. Different cytokines, chemokines, prostaglandins, reactive oxygen species, and transcription factors present in the microenvironment of these chronic oral inflammatory conditions are known mediators of cell proliferation, epithelial-to-mesenchymal transition, and invasion [12,15].

The pathogenesis of OSCC is complex; it includes genetic predisposition, risk factors and interactions of all the components of the immune system. Interleukin-17 has been recently marked as a key link between inflammation, wound healing, and cancer [16] and its role in OSCC development and progression is the focus of this review.

The risk factors associated with OSCC include alcohol, marijuana, and tobacco (including cigarettes, pipes, smokeless tobacco, vaping) consumption, chewing of betel leaf and areca nut, human papillomavirus (HPV) infection, and prolonged oral dysbiosis [17]. Alcohol and tobacco consumption precede the development of 75% of all OSCC cases [18], with increased risk when both are used on a regular basis [19]. Cigarette smoke, by stimulation of interleukin-(IL-)17 mediated inflammation, induces genomic instability and thus can play an important role in the cancer development [20]. Moreover, it is known that hepatic steatosis, inflammation, fibrosis, and finally hepatocellular carcinoma (HCC), induced by alcohol are critically regulated by IL-17A [21].

About 15–20% of all OSCCs are related to high-risk HPV infection [22], with the most predilected localizations in the oropharynx, tonsils, and base of the tongue [23]. Almost 70% of OSCCs localized in the oropharynx are positive for high-risk HPVs [24]. The improved prognosis of HPV-positive OSCC has been shown to be associated with higher numbers of tumor infiltrating T helper (Th)17 cells and lower numbers of IL-17 producing non-T cells [25]. The broken balance between more than 700 bacterial species that are part of the bacterial flora in the oral cavity causes dysbiosis and leads to development of oral diseases such as periodontal disease [26]. Close links between the composition of the oral bacterial flora, presence of specific oral bacteria and OSCC have been reported [27,28,29]. Furthermore, several studies have shown that oral bacteria such as *Porphyromonas gingivalis* and *Fusobacterium nucleatum* alter the oral microbiota in a way that enhances the risk of OSCC development and OSCC progression by enhancing cell proliferation, inhibiting apoptosis, and improving tumor invasion [30,31,32]. These pathogens also activate monocytes resulting in increased IL-17 production by human immune cells, a process that precedes development of periodontal diseases [33] that can be a risk factor for OSCC.

A recent meta-analysis reported dominant overexpression of IL-17 and Th17 cells in the local inflammatory infiltrates in oral lichen planus, predisposing inflammatory condition to OSCC, higher concentration of IL-17 in the serum of these patients, and more intense IL-17 expression in erosive than in reticular oral lichen planus, suggesting a positive correlation between IL-17 levels and disease severity [34].

Various polymorphisms and combinations of specific genetic mutations have been reported to be associated with an increased risk for development of OSCC [35]. Interleukin 17A and IL-17F polymorphisms are associated with increased risk for OSCC and are related to tumor stage and differentiation. In addition, it has been shown that the IL-17A and IL-17F polymorphisms increase the risk of OSCC developing in a population exposed to two other risk factors, smoking and alcohol [36].

Development and progression of OSCC are strongly affected by different components of the immune system [37]. However, the impact of IL-17, an inflammatory cytokine that closely contributes to the development, progression and metastasis of various tumors and affects the sensitivity to chemotherapy and radiation therapy on OSCC formation and progression requires further elucidation. In this review, the possible roles of IL-17 in OSCC development will be discussed.

## 2. Interleukin-17, IL-17R, and Signal Transduction

Interleukin-17 is an inflammatory cytokine with proven roles in chronic inflammatory and autoimmune diseases [38] and in the formation and progression of cancers of the colon [39], stomach [40], pancreas [41], liver [21,42,43], skin [44], lung [45], and myeloma [46]. Interleukin-17as an inflammatory cytokine helps in establishing the tumor stroma that supports tumor formation and growth. Interleukin-17is a key cytokine of CD4+ T helper 17 (Th17) cells [47]. In addition to Th17 cells, a number of immune cells also produce IL-17, including γδ T cells [48], cytotoxic T cells (CD8+ αβ T cells [49] natural killer (NK) cells [50], invariant (i)NKT cells [51], innate lymphoid cells [52], neutrophils [53], eosinophils [54], and macrophages [55]. These cells are collectively named type 17 cells [56]. The transforming growth factor (TGF)-β together with IL-6 drives the differentiation of Th17 cells [47]. Most type 17 cells produce IL-17 after stimulation with IL-1 and IL-23 [57,58,59,60], which activates the transcriptional factors Signal transducer and activator of transcription (STAT)3 [61] and RAR-related orphan receptor (ROR)γt [62]. In γδT cells, the expression of IL-17 is controlled by the transcription factor c-Maf [63]. All these type 17 cells play spatially and temporally specific roles in physiological responses, but in chronic inflammatory conditions and cancer, all of them produce IL-17 that has roles in the maintenance of pathological processes [64].

**Figure 1:**
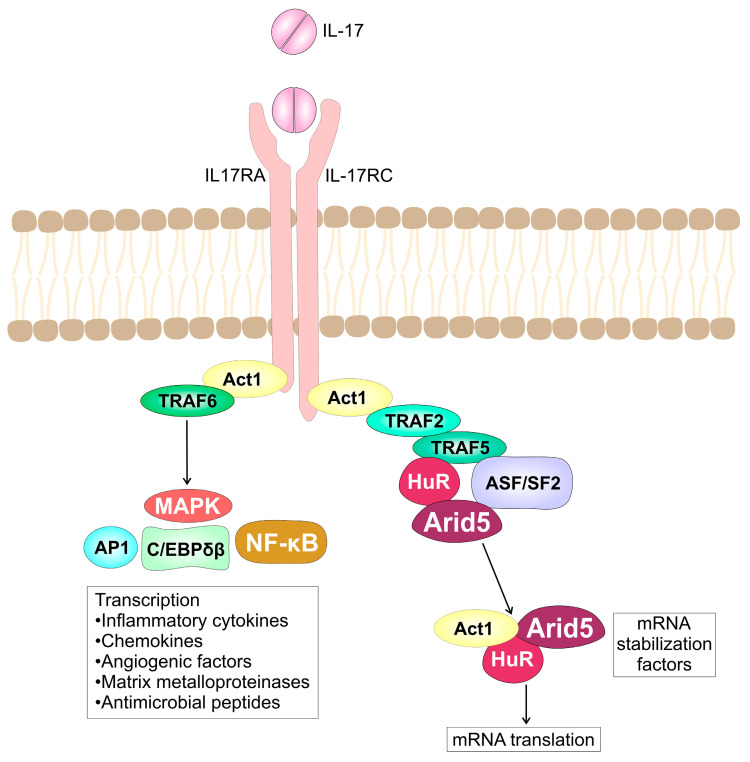
Interleukin-17 signaling. The IL-17R mediated transcriptional and posttranscriptional changes in target cells.

The family of IL-17 cytokines consists of six cytokines: IL-17A (the prototype of IL-17), IL-17B, IL-17C, IL-17D, IL-17E (also known as IL-25), and IL-17F. The most studied cytokines of the IL-17 family are IL-17A and IL-17F, the two cytokines with the highest homology that are usually co-produced [65]. Interleukin-17A and IL-17F exist either as homodimers or as a heterodimer, and all forms of the cytokine signal through a heterodimeric dimeric receptor complex consisting of the IL-17 receptor A (IL-17RA) and IL-17RC [66]. All types of IL-17R molecules contain a conserved cytoplasmic motif known as the similar expression of fibroblast growth factor and IL-17R (SEFIR) domain [67]. The cytosolic adaptor Act1 contains a SEFIR domain and interacts with IL-17RA and IL-17RC through homotypic SEFIR interactions leading to the activation of all IL-17-dependent signaling pathways [68,69]. In addition to the SEFIR domain, IL-17RA also contains a non-conserved region that extends ~100 residues beyond the SEFIR, termed a “SEFIR-Extension” (SEFEX) that is required for IL-17RA signaling [70,71], and with SEFIR comprises a single composite structural motif [72]. Interleukin-17 upregulates the expression of signature genes (inflammatory cytokines, chemokines, antimicrobial peptides, and matrix metalloproteinases) either by inducing de novo gene transcription or by stabilizing target mRNA transcripts. The earliest event after IL-17 receptor engagement is the association of the IL-17R with Act1, a key adaptor molecule required for both the transcriptional and post-transcriptional changes induced by IL-17 (Figure 1) [73]. The Act1 is a nonredundant activator of IL-17RA dependent signals. It functions as a Lysine-63 (K63) E3 ubiquitin ligase, which recruits and ubiquitinates TNF receptor associated factor 6 (TRAF6), leading to the recruitment and activation of the transforming growth factor β-activated kinase (TAK)1 and the inhibitor of nuclear factor (NF)-κB kinase (IKK) complex [74,75]. The IKK then phosphorylates the IκB subunit of the NF-κB:IκB complex and marks it for proteasomal degradation, exposing thus a nuclear localization signal on NF-κB, and allowing the rapid nuclear translocation and consequent inflammatory gene transcription [76]. Additionally, IL-17 is able to activate the spleen tyrosine kinase (Syk) tyrosine kinase associated with IL-17RA, Act1 and TRAF6 in keratinocytes, resulting in activation of NF-κB [77]. The C/EBPs CCAAT/enhancer-binding protein (C/EBP) transcription factors are additional transcriptional regulators activated by IL-17 [78]. The TRAF6 activated by IL-17 binding to IL-17 receptor promotes activation of mitogen-activated protein kinase (MAPK) pathways: extracellular signal-regulated kinase (ERK), p38 and JUN N-terminal kinase (JNK) [79], AP1 (activator protein 1) pathways, and the C/EBPβ transcription factors [74]. The IKK mediates p105 phosphorylation, releases TPL2 kinase from p105 and activates p38 and JNK [80]. Furthermore, IL-17 induces formation of a multi-protein signaling complex that comprises IL-17R-ACT1-TRAF4- mitogen-activated protein kinase kinase kinase (MEKK)3-MEK5, which activates extracellular signal-regulated kinase (ERK)5, but not NF-κB, p38, JNK, or ERK1/2, inducing expression of IL-17 target genes, which leads to keratinocyte proliferation and eventually tumor formation [81].

Transcriptional changes induced by IL-17 are relatively weak in contrast to less well-defined but more robust IL-17 induced posttranscriptional changes that include stabilization of specific mRNAs and protein translation. The mRNAs that encode inflammatory mediators are relatively unstable, enabling the fine-tuning of gene expression during inflammatory responses [82]. Interleukin-17 enhances the level of inflammatory mRNAs by protection of inflammatory mRNAs from degradation through the inhibition of Regnase-1, an endoribonuclease [83]. The adaptor for IL-17R, Act1, can also function as an RNA binding protein in the complex with TRAF2 and TRAF5, and thus interacts with target mRNAs, including *C-X-C Motif Chemokine Ligand 1* (*Cxcl1)*, *Colony stimulating factor-2* (*Csf2)* and *Tumor necrosis factor* (*Tnf)* [83], playing a direct role in mRNA metabolism, stabilization, and translation. The SEFIR domain of Act1 recognizes and binds to SEFIR-binding elements in IL-17 target transcripts, enabling the Act1 direct formation of three compartmentally distinct protein–RNA complexes that prevent mRNA decay in the nucleus, inhibit mRNA decapping in P-bodies, and promote client mRNA translation in the polyribosomes [83]. Interleukin-17 also induces the interactions of Act1 and IKKi and TANK-Binding Kinase 1 (TBK1), which translocate to nucleus and phosphorylate splicing factor(SF)2 and diminishes SF2 mediated mRNA decay [83,84]. Furthermore, Act1 facilitates the binding of HuR to mRNA, enabling the movement of mRNAs into polyribosomes for translation [85]. Interleukin-17 also induces the expression of the RNA binding protein, Arid5a, which binds TRAF2 and stabilizes IL-17 induced transcripts by competing with Regnase-1 [84]. The post-transcriptional regulation of mRNA by IL-17 is part of a self-reinforcing mechanism that potentiates IL-17 activity [86]. Moreover, the ability of IL-17 to modulate the post-transcriptional mRNA metabolism can explain its strong proinflammatory actions in vivo in contrast to modest transcriptional activation in vivo [64].

The expression of IL-17 receptors is ubiquitous, but the main targets of IL-17 are non-hematopoietic cells [87]. Interleukin-17 signaling induces the production of proinflammatory cytokines (IL-1, IL-6, G-CSF, GM-CSF, and TNF-α), chemokines (CXCL1, CXCL2, CXCL5, CCL2, CCL7, CCL20, and IL-8), matrix metalloproteinases (MMP1, MMP3, MMP9, and MMP13), and anti-microbial peptides (β-defensins, S-100 proteins) [88]. The biological activities of IL-17 are often the result of synergistic or cooperative effects with other inflammatory cytokines such as TNF-α [89] leading to amplifying of inflammatory response. Interleukin-17 also cooperates with other cytokines IFN-γ, IL-13, TGF-β, and microbial products [90,91,92].

## 3. IL-17 Dependent Inflammation and OSCC

Dysregulated IL-17 is marked as a major pathogenic factor involved in both the early and late development stages of various cancers [16]. The role of IL-17 in potentiation of OSCC development has been shown in the mouse model. Expression of IL-17 and IL-17-induced inflammatory molecules has been significantly upregulated during progression from normal mucosa to hyperplasia and tumor formation, while inhibition of IL-17 delayed the development of precancerous and cancerous lesions in mice treated with 4-Nitroquinoline 1-oxide (4NQO), and prolonged their survival [93].

In support of results obtained in animal studies, the serum levels of IL-17A [94] and IL-17F [95] were significantly higher in OSCC patients when compared to controls. One study has reported a lower concentration of IL-17F in the serum of OSCC patients compared to controls that was positively associated with the numbers of CD3+CD4+ T cells, indicating that CD4+ T cells are the main source of IL-17F during the development of OSCC [96]. Furthermore, concentrations of IL-17A, IL-17F, and TNF-α have been reported to be significantly higher in saliva of patients with cancer of the oral cavity and oropharynx and are strongly associated with disease advancement [97]. Polymorphisms of IL-17 and IL-17F have been associated with oral squamous cell carcinoma risk, and are related to tumor stage and differentiation, and potentiate the protumorogenic effects of tobacco and alcohol, enhancing thus the risk of OSCC development [36]. Recent analysis has revealed that the most significant differentially expressed genes in OSCC are, among others, genes encoding molecules involved in the IL-17 signaling pathway [98].

Several studies have reported the presence of IL-17 and IL-17 producing cells in the peripheral blood of OSCC patients. Significantly higher frequency of Th17 cells in the peripheral blood of OSCC patients compared to controls has been reported [99]. These cells were found to express markers of activation and CCR6 chemokine receptor. The cytokine profiling of these cells revealed three Th17 subsets (Th17/1 (IL17A+IFNγ+), Th17/inflammatory (IL17A+IL8+), and Th17/2 (IL17A+IL4+)), which all were elevated in OSCC patients compared to controls [99]. A shift toward the Th17/1 cell type was observed in the early stage OSCC patients [99]. In line with this, an increase in the Th17/Tregs ratio in early stages of OSCC without lymph node involvement and a decrease in this ratio in higher clinical stages and lymph node involvement has been reported, indicating Th17 and Tregs cells as significant prognostic factors in OSCC patients [100]. Additionally, significantly higher frequencies of Th17 and IL-17 producing CD8+ cells (Tc17) were found in the peripheral blood of head and neck cancer patients that were positively correlated with the disease stage, suggesting the role of IL-17 in the creation of the inflammatory pro-tumor environment [101].

A significantly higher expression of IL-17 in OSCC tissue and tumor margins compared to normal tissue, detected by immunohistochemical staining, has been reported [102]. Furthermore, a significant correlation between IL-17 positive tumor budding (insulated single or small clusters of cancer cells (no more than five cells) that indicates the loss of cellular cohesion and the presence of active invasive movement [103]) and tumor classification, lymph node metastasis, distant metastasis, clinical stage, and OSCC recurrence was found [102]. Tumor budding at the tumor invasion front of OSCC is related to local metastasis and poor prognosis [104]. The presence of IL-17 in OSCC budding suggests that IL-17 takes a role in tumor invasion and promotes OSCC progression. In another study, increased expression of IL-17 in the OSCC tumoral islands, the tumor-stroma interface, and more distant stroma was observed [105]. Helper T cells, cytotoxic T cells, and macrophages were identified as the main cellular source of IL-17 in this study and there was no IL-17 in supernatants of the OSCC cell lines [105]. As tumor cells in OSCC tissue express IL-17R [101], it is very possible that IL-17 released by immune cells in the OSCC microenvironment directly stimulates OSCC tumor cells and maybe induces their proliferation because it was shown that IL-17 stimulates proliferation of OSCC cell lines in vitro [101]. Analysis of tumor infiltrating lymphocytes in OSCC revealed higher amounts of Th17, Tc17, and Tregs in tumor tissue, ones higher in comparison with their frequency in peripheral blood, and a correlation of high Th17/Treg ratio and overall survival [106]. Furthermore, it has been found that the frequency of IL-17+ T cells was inversely correlated with tumor size, while the frequency of Foxp3+T cell in tumor infiltrates was positively correlated with the TNM stage [106]. Lee et al. reported increased prevalence of IL-17 producing FOXP3+CD4+ tumor infiltrating lymphocytes in oral squamous cell carcinoma that showed suppressive capacity [107]. These cells express CCR6 and suppress the proliferation of autologous CD4+CD25- responder T-cells in vitro [107]. In fact, both Th17 and Tregs were accumulated in the tumor microenvironment at early stages of the OSCC development, but in parallel with tumor progression, the numbers of Th17 cells in the infiltrates gradually decreased while the Treg numbers increased in infiltrates as the disease progresses. The reason for these opposite results may be the high plasticity of the Th17 cells that can convert into Th1, Treg, or Th2 cells in response to various microenvironments and thus gain various contrary activities [108]. Recently it has been reported that in the late stage of 4NQO induced oral tumors in mice, ablation of Treg cells triggers an increase in the number of both CD4+ and CD8+ effector T cells within oral lesions [109]. Interestingly, this manipulation does not induce tumor regression, instead it induces the effector T cell dependent rapid emergence of invasive OSCC [109].

Chronic IL-17 activity induces the production of proinflammatory cytokines and chemokines and thus stimulates accumulation of neutrophils in the blood and tissues and induces inflammation, it mediates the release of pro-angiogenic cytokines from fibroblasts stimulating wound-healing pathways [16] and may be associated with tissue destruction through matrix metalloproteinases [110,111]. All these processes may play a role in OSCC formation and progression. Dysregulated IL-17 production can be triggered by a pathogenic microbiota, which induces continuous activity of the immune system to limit invasive colonization. It has been recently shown that experimental periodontitis promotes the formation of OSCC [112]. The oral microbiota in periodontitis directly activates IL-17+γδ T cells, stimulates signal transducer and activator of transcription 3 (STAT3) pathway, and promotes infiltration of oral carcinoma tissue with M2-tumor-associated macrophages [112]. Inhibition of γδ T cells led to decreases in the concentration of IL-17A, the phosphorylation level of STAT3, and the size of tumors [112]. Moreover, the proportion of IL-17+ γδ T cells and the phosphorylation of STAT3 were higher in the tissues obtained from OSCC patients with periodontitis group compared to OSCC patients without periodontitis [112]. This study provides experimental evidence regarding cross talk among the microbiota, IL-17 signaling, inflammation, and oral carcinoma cells.

The significance of IL-17 in OSCC progression can be indirectly confirmed by the results of the study that showed overexpression of Akt1, nonredundant activator of IL-17RA-dependent signals in OSCC tissue, association of genetic alterations of Akt1 with a poor clinical outcome in OSCC, and decreased expression of proteins regulating cell survival leading to decreased OSCC cell survival after silencing of Akt1 [113].

The IL-23R knockout mice had faster progression of premalignant oral lesions to cancer, compared to wild type mice, but both groups developed the same histological OSCC score 18 weeks after initiation of 4NQO treatment [114]. Although IL-23R KO mice had reduced inflammation (lower levels of IL-17 among other inflammatory cytokines) in the stage of premalignant lesions, conversion to the inhibitory phenotype of inflammatory cells (IL-10 producing) in the stage of oral cancer lacked inflammation. Results of this study are in line with previously listed results obtained from human samples and confirm the importance of IL-17 in conversion of premalignant lesion to oral cancer, but also show the importance of IL-23 signaling and, indirectly, Th17 cells in limiting OSCC growth [115]. Mice bearing premalignant oral lesions treated with a TGF-β type 1 receptor inhibitor plus IL-23 in order to sustain the Th17 phenotype also slowed the progression of premalignant lesion to cancer [116]. Protective roles of Th17 and Tc17 cells in eradicating already established tumors have been reported [116].

Due to the impact on mRNA metabolism, IL-17 is able to perform its activity synergistically with other cytokines and activates diverse signaling pathways. Interleukin-17 and TNF-α synergistically activate NF-κB, a transcriptional factor with known roles in the promotion of inflammation and OSCC progression [117,118,119]. Interleukin-17 signals cooperatively with the IFN-γ enhances activation of STAT1, a molecule involved in signaling pathways important for OSCC growth and metastasis [120,121]. Furthermore, IL-17 cooperates with TGF-β and activates SMAD signal transducers known for their modulation of OSCC microenvironment and promotion of OSCC invasion [122,123]. Further investigations are needed in order to determine the impact of different synergizing partners of IL-17 in driving the inflammatory pathways and the OSCC outcome.

## 4. The Role of IL-17 Stimulated Microenvironmental Cells in OSCC Progression

**Figure 2:**
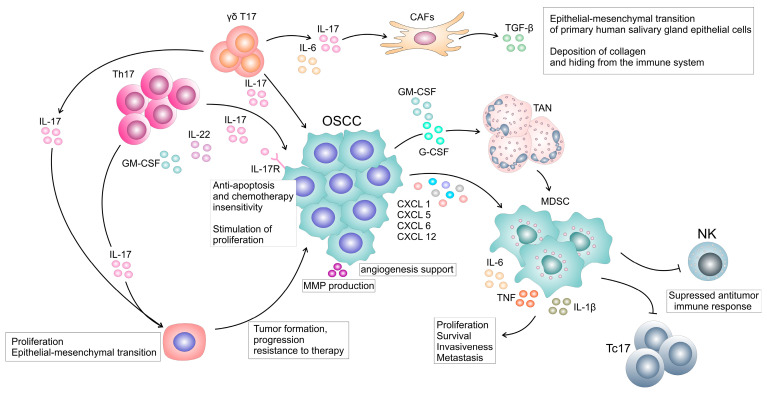
The roles of IL-17 induced mediators in OSCC pathogenesis. Interleukin-17 from γδ T cells Th17 cells could stimulate proliferation of oral epithelial stem cells and their transformation which could be the initiating factor in OSCC pathogenesis. These could also contribute to progression of already established tumors and to therapy resistance. Interleukin-17 directly stimulates proliferation of tumor, prevents apoptosis leading to chemotherapy insensitivity. Moreover, IL-17 stimulates the production of G-CSF and GM-CSF, cytokines that expand, and chemokines that recruit myeloid cells, and neutrophils or granulocytic myeloid derived suppressor cells (MDSCs). These myeloid cells produce various angiogenic factors, MMPs, and promote tumor progression by stimulation of proliferation, survival, invasiveness, and metastasis and by suppressing antitumor immune activity of Tc17 and NK cells. Interleukin-17 stimulates cancer associated fibroblasts (CAFs) that produce mediators which promotes epithelial to mesenchymal transition and deposition of collagen resulting in escape from immune response. In addition, IL-17–induced protumoral cytokines, such as IL-6, function in a paracrine manner to enhance tumor growth and survival.

Interleukin-17 activates transcriptional factors and stabilizes specific mRNAs resulting in the production of inflammatory mediators and various ligands that induce favorable microenvironment for OSCC progression (Figure 2). Extracellular vesicles (EVs) contain proteins and nucleic acids that efficiently mediate intercellular communication [124] and are considered as one of the main players in the communication between cells in the inflammatory tumor microenvironment [125], actively contributing to tumor growth, invasion, and metastasis [126]. Recently it has been shown that OSCC-derived EVs induce overactivation of the IL-17A-signaling pathway in tumor tissue, causing an inflammatory cytokine imbalance in the tumor microenvironment, and thus promote OSCC xenograft tumor growth [127].

### 4.1. Impact of IL-17 on Myeloid Cells

Recruitment of neutrophils is critically controlled by IL-17 and contributes to host defense [128], but a sustained IL-17 activity because of non-resolving inflammation and associated chronic wounding, persistent infection, or carcinogenesis, induces generation of pathogenic myeloid cells, and myeloid derived suppressor cells (MDSC) [129]. Interleukin-17 stimulates G-CSF production that promotes the expansion of granulocytes [130], and stimulates production of the proinflammatory cytokines IL-6 and TNF-α that play roles in inducing a suppressive phenotype in the recruited myeloid cells [131]. The MDSCs suppress the anti-tumor functions of T and natural killer (NK) cells and promote tumor cell proliferation, survival, invasiveness, and metastasis [132].

Two studies have reported higher levels of MDSC in the peripheral blood of OSCC patients compared to controls, with significant positive correlation with the tumor size and stage [133,134]. In addition, the levels of MDSC are shown to correlate with tumor progression in human head and neck cancer [135]. Moreover, granulocytic/polymorphonuclear MDSC has been detected in OSCC tissue accompanied with increased levels of Th17 cells in peripheral blood and tumor tissue [136]. Another study has reported higher percentages of peripheral blood MDSCs and Th17 cells, and the level of IL-17 in serum of OSCC patients compared to healthy controls [137]. Additionally, significant correlation was found between the number of MDSCs and the level of IL-17, while no correlation was found between the numbers of MDSCs and Th17 cells implying that other cells, not Th17, are the main source of IL-17 in OSCC [137]. Infiltration with neutrophils and elevated expression of TGF-β1 and IL-17A in OSCC tissues accompanied with increased expression of MMP9 and decreased expression of CCL3 in circulating neutrophils, has been reported [138]. The cooperative effects of TGF-β1 and IL-17A were also reported in this study. It was shown that neutrophils in vitro exposed to TGF-β1 and IL-17A have augmented protumor activities that induced cell migration, proliferation, invasion, stemness, and epithelial to mesenchymal transition in OSCC cells in vitro [138]. Combined positivity for tumor associated neutrophils, MMP-9, IL-17, and CD105 was reported to be associated with the metastasis-prone phenotype of OSCC [139].

In a mouse model of oral cancer induced by 4NQO, higher number of MDSCs in the peripheral blood and spleen during carcinogenesis was reported in two studies [140,141]. Depletion of MDSCs in the mouse model of 4NQO induced oral cancer significantly ameliorated carcinogenesis that was promoted by a high fat diet [142]. The importance of MDSCs in 4NQO carcinogenesis was confirmed by the study that shows the difference in tumor growth between CD24−/− and CD24+/− mice was blunted by immuno-depletion of MDSCs [142]. Furthermore, *Porphyromonas gingivalis* induced progression of oral cancer by generating a cancer-promoting microenvironment that contains increased number of MDSCs [143,144]. In addition, it has been shown that *C. albicans* promotes development of 4NQO-induced oral cancer via the IL-17A/IL-17RA induced tumor associated macrophages [145]. Reduction in 4NQO induced lesions upon treatment with anti-PD-1 monoclonal antibody in vivo was accompanied by reduction in MDSCs in the lesion-microenvironment and peripheral lymph nodes [146]. The therapeutic effects of anti-hypoxic agents in 4NQO oral cancer have been accompanied by reduced presence of MDSCs in the tumor microenvironment [147].

Collectively, these studies demonstrate that myeloid suppressor cells that are IL-17 dependent accumulate in oral squamous cancers and contribute to tumor progression.

### 4.2. Impact of IL-17 on Cancer Associated Fibroblasts

One of the constituents of the tumor microenvironment is cancer associated fibroblasts (CAFs) that play one of the main roles in cancer progression. An additional contribution of IL-17 to forming the microenvironment that supports tumor growth is tumor immune exclusion by affecting CAFs to increase deposition of extracellular matrix [148]. Interleukin-17 contributes to pathological fibrosis in many organs including the lung and liver, enhances pro-fibrotic phenotypes in synergy with TGF-β [149], and in synergy with IL-22 contributes to the epithelial-mesenchymal transition in primary human salivary gland epithelial cells that are isolated from healthy subjects [150]. Deletion of IL-17 signaling, specifically in CAFs in late-stage tumors, led to reduced proliferation and numbers of CAFs, and reduced collagen deposition in murine models of cutaneous squamous cell carcinoma [148]. This reduction in CAFs and extracellular matrix made the tumors susceptible to anti–PD-L1 therapy [148]. There are many studies on clinical specimens showing that CAFs play a role in OSCC proliferation, susceptibility to antitumor immune response, progression, invasiveness, resistance to therapeutics [151,152,153,154,155]. Therefore, future studies in animal models are needed to elucidate the role of IL-17 signaling in CAFs in OSCC.

## 5. Protective Roles of IL-17 in Oral Squamous Cell Carcinoma

Interleukin-17 was reported to play protective roles in several types of cancer, including oral, colon, and hepatocellular carcinoma [95,155,156]. Several articles reported protective roles of IL-17F in OSCC. Most cells derived extracellular IL-17F at the tumor invasion front, which was associated with better disease-specific survival among patients with all stages oral tongue squamous cell carcinoma [157]. Two studies reported decreased serum level of IL-17F in OSCC patients compared with healthy controls [95,96]. Antitumor effects of IL-17F against OSCC cells were observed in vitro. Interleukin-17F inhibited the proliferation and random migration of oral tongue squamous cell carcinoma cells, HSC-3 [158]. Furthermore, IL-17F suppressed the human umbilical vein endothelial cells tube formation suggesting antiangiogenic effects of this cytokine [158]. Additionally, IL-17F suppressed cancer associated fibroblasts mediated invasion of oral tongue squamous carcinoma cells in tumor spheroids [158]. Recently, the potential of IL-17F to inhibit the formation of vasculogenic mimicry structures, an alternative vasculogenic system made by aggressive tumor cells implicated in treatment failure and poor survival of cancer patients, was reported [159]. One study also reported the protective role of IL-17A in OSCC, mediated by promotion of Th17 differentiation associated with suppressed growth of implanted OSCC tumors in nude mice [160].

## 6. Direct Effects of IL-17 on Tumor Cells

It has been shown that IL-17 stimulation, by activating mitogenic signaling pathways, can directly promote the proliferation of keratinocytes and intestinal epithelial cells [161,162]. Interleukin-17 mediated signaling is critical for the maintenance and repair of tissue barrier function in the oral cavity [163]. The main producers of IL-17 in the oral mucosa are γδT cells that release this cytokine spontaneously as part of the process of maintaining epithelial integrity and also under inflammatory conditions [163]. Since recently, it is known that IL-17 can directly promote the proliferation of premalignant cells, that play a crucial role in the early stage of tumorigenesis. Interleukin-17 is a critical inflammatory signal that activates a group of Lrig1+ stem cells that normally reside in the hair follicle to participate in wound healing in the skin [164]. Interleukin-17 induced transactivation of EGFR, Src, and ERK5 in vivo that leads to expansion and migration of Lrig1+stem cells and their transformation, links IL-17 mediated wound healing and tumorigenesis [164]. The negative regulator of EGF, Lrig1, induced EGFR activation [165], but IL-17 is able to transactivate EGFR and activates cells in a state of inflammation or environmentally challenged. Oral epithelial stem cells that express Lrig1 are slow-cycling but are stress responsive [166], thus it is possible that IL-17, in the states of chronic inflammation in the oral cavity and precancerous lesions, such as lichen planus where IL-17 expression in the lesion correlates with disease severity [34], stimulates these cells for tissue repair and possibly induces tumorigenesis. Recently it was shown that extracellular vesicles obtained from mouse OSCC cell lines increased serum levels of IL-17 in mouse recipients and significantly increased xenograft tumor growth and invasion [167]. Furthermore, treatment with extracellular vesicles significantly enhanced the expression levels of crucial molecules in the IL-17A pathway, IL-17A, TRAF6 and c-FOS in tumor tissue and suppressed immune responses of CD8+ cells in vivo [167]. Interleukin-17A showed direct effects on OSCC cells in vitro, it enhanced cell migration and invasion in SCC15, a tongue squamous cell carcinoma cell line [168].

## 7. IL-17 and Cancer Therapy

Intratumoral inflammatory mechanisms are one of the most influential players in development of resistance to therapy [169]. Since IL-17 plays the main role in developing the protumorigenic inflammatory environment and drives tissue repair it is expected that it also contributes to tissue healing after chemotherapy and radiotherapy and its role in causing chemoresistance is being explored. Since IL-17 induces the activation, proliferation, and epithelial-to-mesenchymal transition of quiescent Lrig1 expressing epithelial stem cells [165], and Lrig1 can be induced in cancer cells, IL-17 signaling could provide better survival of tumor cells after chemo- or radio-therapy. The low-dose irradiation induces expression of IL-17 in tumor beds and enhances the growth of subsequently implanted tumor cells, while treatment with anti-IL-17 antibody abolished the acceleration of tumor growth, confirming the key role of IL-17 in enhancing tumor growth in pre-irradiated tumor beds [170]. Interleukin-17 plays a role in restoration of the damaged epithelia in radiation induced oral mucositis and attenuates epithelial damage [171], which imply that a similar IL-17 mediated process could contribute to better recovery of OSCC cells after radiotherapy and eventually chemotherapy and subsequently contribute to resistance of oral squamous cancer to therapy. Flavonoid induced better sensitivity of chemo-resistant OSCC cells to cisplatin is accompanied by the downregulation of IL-17 [171]. Moreover, in the mouse model of OSCC, it has been shown that inhibition of IL-17A combined with the PD-1 blockade delayed the development of precancerous and cancerous lesions and prolonged the survival of 4NQO-treated mice, suggesting the IL-17A blockade as a potential approach to augment the tumor eliminating effects of anti-PD-1 therapy [92].

## 8. Conclusions

In this review, the literature regarding the role of IL-17 in the promotion of OSCC tumorigenesis is summarized. Interleukin-17 can promote OSCC tumorigenesis by several pathways, such as provisioning the microenvironment that promotes cell transformation and tumor formation and inducing production of molecules (VEGF, MMP-9, MMP-13, CCR6, and PGE2) that promote invasiveness and angiogenesis; enhancement of immunosuppressive effects of MDSCs that promote tumor cell proliferation, survival, invasiveness and metastasis; direct proliferative effects on tumor cells; activation and transformation of stem cells. In contrast, the results of some studies mark IL-17 as a protective cytokine in OSCC. These opposite reports could be explained by different phases of the disease when the evaluation of cytokine was done. In addition, the cellular source of IL-17 could be related to its impact on tumor formation and proliferation. Therefore, further studies are needed for better elucidation of the role of IL-17 in OSCC tumorigenesis, exploring synergizing partners of IL-17 in driving the inflammatory pathways in OSCC, and the role of IL-17 stimulated CAFs in OSCC pathogenesis.

There is limited information regarding the role of IL-17 in regulating the response to checkpoint inhibitors or other immunomodulators and to chemotherapy and radiotherapy of OSCC. It is important to further explore whether blockade of IL-17 sensitizes resistant oral squamous cell carcinomas to chemo-, radio- and immuno-therapy, or can it be used in the prevention of OSCC.

## Data Availability

This study is a review article and does not generate any new datasets or involve the use of existing datasets. The authors have drawn upon and analyzed previously published research in the field, and all relevant sources have been cited within the manuscript. Readers interested in accessing the original data sources can refer to the cited references for further information.
